# Trigger tool versus verbal inventory to detect surgical complications

**DOI:** 10.1007/s00423-015-1337-4

**Published:** 2015-09-10

**Authors:** A. Visser, A. E. Slaman, C. M. van Leijen, D. J. Gouma, J. C. Goslings, D. T. Ubbink

**Affiliations:** Department of Surgery, Academic Medical Center, University of Amsterdam, H1-213, Meibergdreef 9, 1105 AZ Amsterdam, The Netherlands

**Keywords:** Complication, Registration, Predictor, Surgery, Trigger tool

## Abstract

**Purpose:**

Traditionally, registering complications after surgery is based on voluntary reporting or incident reports. These methods may fail to detect the total number of complications. A trigger tool was developed to detect complications in hospitalized surgical patients. In this diagnostic study, we compared its sensitivity and specificity with the verbal inventory by surgical staff and residents.

**Methods:**

A set of 31 potential triggers was chosen based on a systematic review and availability in hospital databases. The trigger tool was developed using multivariable regression and Receiver Operating Characteristic (ROC) analyses. A reference standard consisted of 300 patients, 150 with and 150 without complications. Sensitivity and specificity of the trigger tool and verbal inventory were determined.

**Results:**

The final trigger tool consisted of nine triggers. Sensitivities of the trigger tool and verbal inventory were 70.7 vs. 78.7 %, respectively, while specificities were 70.0 vs. 100.0 %, respectively. Sensitivity values to detect major complications were 97.2 vs. 80.6 %, respectively.

**Conclusions:**

The proposed customized trigger tool for a university hospital to detect surgical patients with complications appeared as accurate as a verbal inventory and even more accurate to detect major complications.

## Introduction

Registration of surgical complications is important to assess and improve quality of surgical care [1]. Also, analyzing surgical complication registration outcomes can and should lead to improved patient outcomes [2]. Despite widespread acknowledgement that complications should be reduced, controversy exists how to detect and record these complications [3].

Traditional efforts to detect complications have focused on voluntary reporting or incident reports [4]. These methods have often been poorly successful in the detection of complications [3]. For example, registration during verbal hand-off meetings yields a registration rate of only 86 % of all complications [5]. Moreover, to achieve adequate reporting of complications, a sufficient number and diversity of surgeons should participate in the daily verbal hand-off meetings, but this is time-consuming for highly qualified surgeons. Hence, hospitals would benefit from a more effective way to identify complications and to complete their registration.

An attempt to design a more uniform, practical, and efficient complication registration method came from the Institute for Healthcare Improvement (IHI), who developed the Global Trigger Tool (GTT) [4]. A “trigger” can be defined as a specific factor that is derived from the patient’s medical record and is associated with an increased risk for complications. These factors can be patient-specific (e.g., lab results, BMI), surgical procedure-specific (e.g., complexity of the procedure), or hospitalization-specific (e.g., length of hospital stay). A “trigger tool” is a set of triggers that identifies patients who are likely to have suffered a complication and thereby indicates which patient records should be checked for complications, for instance by a data manager.

The benefits of (some form of) the GTT to detect complications have been studied in terms of inter-rater reliability among different reviewers on reporting complications^.^ [3, 6–12]. Two studies showed a high specificity (92.0 and 99.0 %), but low sensitivity (23.0 and 28.0 %) [13, 14]. The high specificity means that the methods could be used to replace expensive manual chart reviews because less “falsely positive” charts need to be checked. However, in order not to miss any complications, the sensitivity of the method should also be high.

The aim of this study was to develop a new trigger tool to assess the accuracy and usefulness compared with the verbal inventory.

## Methods

### Patients and setting

This study comprised a model development and diagnostic accuracy study, based on a 1-year sample of hospitalized surgical patients. The study was performed at the department of surgery of a tertiary referral university hospital in Amsterdam. All patients (*n* = 4534) above the age of 17 admitted to or operated by a surgeon from this department between July 2012 and June 2013 were included in this study. This surgical department provides general, gastrointestinal, hepatopancreatobiliary, vascular, and trauma surgical services.

### Verbal inventory and registry of complications

Currently, surgical residents collect preoperative, intraoperative, and postoperative data for each surgical patient real-time. The attending staff may supplement, during the morning hand-off, the complications identified by the residents. Subsequently, the database manager reviews, in retrospect, the charts of the patients identified with a complication for possible additional complications. The data manager uses definitions and agreements about specific clinical situations. In case of uncertainties, the data manager consults the surgeon responsible for the registration. The identification and collection of complications by surgical residents and during the morning hand-off is defined as “verbal inventory.”

All complications are registered and categorized by severity based on the Clavien-Dindo classification in the departments’ complication database [15]. The complication registry categorizes each complication into four grades of severity: grade 1, temporary health disadvantage recovering without reoperation (grade 1 management includes radiological or endoscopic interventions; similar to Dindo grade I, II, and IIIa); grade 2, recovery after reoperation (similar to Dindo grade IIIb); grade 3, (probably) permanent damage or function loss (similar to Dindo grade IV when permanent); and grade 4, death (similar to Dindo grade V).

A “complication” is defined according to national and international standards as “an unintended and unwanted outcome or state during medical care that is so harmful to the patients’ health that it requires (adjustment of) treatment or leads to permanent damage” [16]. Complications that occur after discharge are not registered unless the patient is readmitted within 30 days after discharge.

### Development of the trigger tool

A set of potentially relevant triggers was chosen based on (a) a previous systematic review of the literature in which these triggers were found to be significantly associated with surgical complications [17], (b) questionnaires containing the potential triggers found in the literature answered by 12 surgeons within our hospital to validate or supplement this set of potential triggers, and (c) availability of the trigger in electronic hospital databases. Correctness of the department’s database on complications was monitored by two investigators. Subsequently, two investigators independently extracted the values of the set of potential triggers from hospital databases belonging to the records of patients in the study period. To ensure correctness of the data entry, one investigator (AES) checked a random set of 20 patient records per trigger as entered by the other investigator (CMvL) and vice versa. Univariable logistic regression analysis was performed to find triggers that occurred substantially more often in the group with complications in the departments’ complication database. Variables with *p* < 0.20 were entered into a stepwise multivariable logistic regression analysis to find significant independent triggers (*p* < 0.05). This association was expressed as their odds ratio (OR), 95 % confidence intervals, and *p* values. Continuous variables were dichotomized. Different ranges of cutoff values (based on clinical relevance and literature) were tested for each variable and analyzed with different cutoff limits. The most significant value was set.

#### Reference standard

Of all patients with complications, 86 % were recorded in the current database [5]. In order to validate and compare the trigger tool, a reference standard was formed by a random sample of 150 records with complications and 150 records without complications, meaning oversampling for complications. For each potential trigger, a minimum of 10 records (as a rule of thumb) should be included in the reference standard in a regression analysis. A sample of 150 records was considered to be adequate as a reference standard to be able to detect 15 possible independent predictors.

The medical files were reviewed by two investigators (AES and CMvL). If the investigators did find a complication in the latter 150 records, this record was discarded and added to the records with complications. In case of uncertainties interpreting the texts of the resources about complications, the investigators consulted each other or their supervisors. This procedure was continued until the group without complications also contained 150 verified records.

### Validation of the trigger tool

The independent triggers from the departments’ complication database were subsequently entered in another multivariable analysis, now using the reference standard in order to check their validity. Triggers were kept in the model if they again contributed significantly to the model. If not, we decided to remove the trigger from the model unless the trigger had a low incidence (<10 patients) in the reference standard and was a significant factor in the univariable analysis. The remaining independent triggers formed the final trigger tool.

### Comparing the verbal inventory with the trigger tool

The verbal inventory and trigger tool were compared with the reference standard to calculate their sensitivity and specificity as to the detection of one or more complications and the severity, type, and number of complications registered.

All statistical analyses were performed using IBM SPSS Statistics v.20 (IBM, Armonk, NY, USA).

## Results

### Patients and setting

A total of 4534 patients admitted to the hospital between June 2012 and July 2013 were included in this study. Their mean age was 55 years (range 18–99). Of these, 2529 (55.8 %) were men and 2520 patients (55.6 %) underwent operative treatment. In 795 of the 4534 (17.5 %) patient records, one or more complications were documented in the departments’ database.

### Development of the trigger tool

**Fig. 1:**
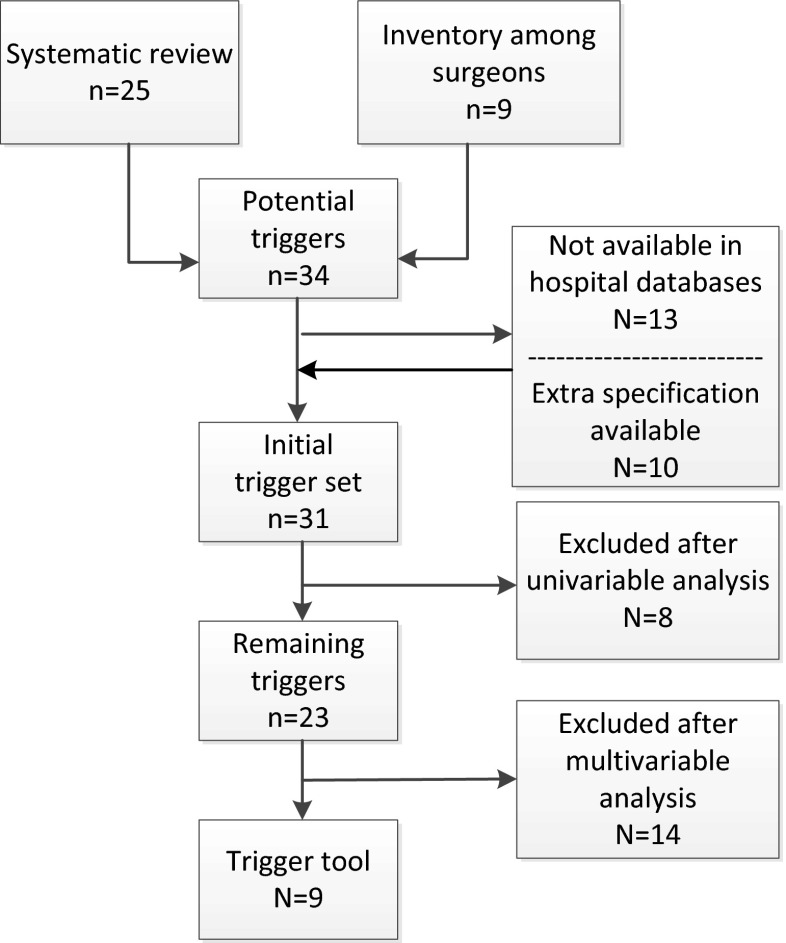
Flow chart of the triggers eventually included in the trigger tool

The systematic review provided 25 potential triggers that were significantly associated with the occurrence of surgical complications [17]. The inventory among the hospital’s surgeons yielded nine additional potential triggers (Fig. 1). This led to a total of 34 potential triggers for data collection. Of these, 21 were readily available in hospital databases and extra 10 specifications of the potential triggers were available (Appendix). For example the trigger emergency operation resulted in two triggers: (1) emergency operation during admission and (2) the highest urgency classification of emergency surgery during admission.

Univariable analysis found 23 out of 31 potential triggers with a *p* <0.20 (Table 1).

**Table 1:** Outcome of univariable analysis of variables possibly associated with complications, expressed as *p* values and 95 % confidence intervals (CI). Study group *N* = 4534

Variables	*p* value	95 % CI	Number of samples
Retrieved from the systematic review			
Sex	0.090	0.749–1.021	4534
*Age (years)*	*<0.001*	*1.007–1.011*	*4534*
BMI	0.678	0.984–1.026	2154
*ASA score*	*<0.001*	*1.239–1.678*	*2141*
MET score	0.963	0.945–1.062	1643
*Emergency procedure*	*<0.001*	*1.873–2.650*	*4534*
Urgency code at moment of admission	0.298	0.881–1.511	4534
*Highest urgency code in admission period*	*<0.001*	*1.470–1.742*	*4534*
*Time required above the scheduled procedure time*	*<0.001*	*1.007–1.011*	*2254*
*DNR*	*<0.001*	*0.309–0.464*	*4168*
Smoking	0.220	0.912–1.491	1956
*COPD/asthma/emphysema*	*0.003*	*1.148–1.990*	*2209*
Hypertension	0.230	0.920–1.412	2288
Increased serum creatinine	0.666	0.734–1.218	1856
*Hyponatremia*	*0.098*	*0.952–1.792*	*1427*
Hypernatremia	0.769	0.417–3.263	1427
*Sodium level outside reference range*	*0.091*	*0.959–1.773*	*1427*
*Increased leukocyte count*	*0.015*	*1.062–1.757*	*1556*
*Decreased serum albumin*	*<0.001*	*2.811–7.382*	*665*
*Use of corticosteroids*	*0.188*	*0.919–1.535*	*4534*
Active alcohol abuse	0.737	0.852–1.120	2066
Retrieved from the inventory among surgeons			
*Surgical procedure (yes/no)*	*<0.001*	*1.966–2.747*	*4534*
*Esophageal resection*	*<0.001*	*4.397–11.171*	*4534*
*Whipple procedure*	*<0.001*	*6.451–18.328*	*4534*
*AAAA*	*<0.001*	*4.574–22.513*	*4534*
*Multi-trauma patient*	*<0.001*	*1.629–5.538*	*4534*
*Length of stay*	*<0.001*	*7.350–10.815*	*4534*
*Admission to ICU*	*<0.001*	*4.290–6.396*	*4534*
*Reoperation*	*<0.001*	*10.917–18.540*	*4534*
Increased C-reactive protein	0.035	1.030–2.270	980
*Complexity of procedure*	*<0.001*	*1.139–1.240*	*2520*

Significant values are presented in italics. Cutoff values: serum creatinine: women >95 μmol/L, men >110 μmol/L; hyponatremia <135 mmol/L; leukocyte count >10.5 × 10^9^ cells/L; serum albumin <35 g/L; use of corticosteroids in 42 days before hospitalization; multi-trauma patient: Injury Severity Score (ISS) >16; increased C-reactive protein >5 mg/L; sodium level outside reference range <135 or >145 mmol/L

*BMI* body mass index, *ASA score* American Society of Anesthesiology score, *MET score* fitness score based on anaesthesiology questionnaire, *DNR* do not resuscitate, *COPD* chronic obstructive pulmonary disease, *AAAA* acute (or ruptured) abdominal aortic aneurysm, *ICU* intensive care unit

Some of the triggers influenced each other, for example, an esophageal resection was associated with admission at the ICU and a higher complexity of surgery. To study their separate effect, we had to develop three models: one general model for patients who underwent a surgical procedure, a second focusing on specific surgical procedures that were prone to result in postoperative complications, and a third for patients who did not undergo surgery, but who could still be burdened with the occurrence of complications during the admission period or in 30 days after discharge (Table 2).

**Table 2:** Multivariable analysis using the departments’ database. Study group *N* = 4534

Trigger	*p* value	OR	Lower 95 % CI	Upper 95 % CI
Model 1				
Length of stay ≥14 days	<0.001	4.948	3.754	6.523
DNR	<0.001	2.177	1.501	3.155
Reoperation	<0.001	7.755	5.384	11.168
Whipple procedure	<0.001	8.201	4.494	14.964
AAAA	<0.001	8.913	1.995	39.816
Esophagus resection	<0.001	4.906	2.818	8.542
Age ≥85 years	0.009	2.944	1.237	7.005
Time required above the scheduled procedure time ≥110 min	<0.001	3.660	2.407	5.562
Model 2				
DNR	<0.001	2.937	2.166	3.982
Time required above the scheduled procedure time ≥110 min	<0.001	4.731	3.307	6.767
Complexity of surgery	<0.001	2.007	1.578	2.552
Urgency operation	<0.001	1.613	1.290	2.016
Model 3				
Length of stay ≥14 days	<0.001	6.399	5.208	7.863
ICU stay	<0.001	2.796	2.226	3.512
DNR	<0.001	2.256	1.799	2.830

*DNR* do not resuscitate, *AAAA* acute (or ruptured) abdominal aortic aneurysm, *ICU* intensive care unit

Multiple models were constructed because of the following: (1) the interference of surgical procedure-specific triggers with other potential triggers and (2) the optimization of significance outcomes when admissions were divided into groups either with or without a surgical procedure during hospitalization

This resulted in 11 independent triggers, 4 of which were continuous variables: length of hospital stay, extension of standard surgical procedure time, complexity of procedure, and age. Their cutoff values were set to the following: length of stay ≥14 days, technical complexity of procedure ≥class 6, age ≥85 years, and time required above the scheduled procedure time ≥110 min. Table 2 shows the results of the trigger tool with the 11 potential triggers again tested in the multivariable analysis, now dichotomized based on these cutoff values.

### Validation of the trigger tool

The triggers were tested using the reference standard (Table 3). The reference standard is not representative for the total population in the departments’ complication database due to oversampling of records with complications. Due to the smaller size of this group, we also studied the incidence of the triggers. The multivariable analysis, now using the reference standard, showed 6 out of the 11 potential triggers to be significant (Table 3). The triggers “time required above the scheduled procedure time ≥110 minutes” and “age ≥85” were found not to be significantly associated with the presence of complications (*p* > 0.20). The incidence of 3 potential triggers was low, but these triggers were significant in the univariable analysis. Therefore, these triggers were nevertheless included in the trigger tool (i.e., esophagectomy (*n* = 6), Whipple procedure (i.e., pancreatoduodenectomy; *n* = 7), and abdominal aortic aneurysm surgical procedure (*n* = 6)).

**Table 3:** Multivariable analysis using reference standard. Study group *N* = 300

Trigger	*p* value	OR	Lower 95 % CI	Upper 95 % CI
Model 1				
Length of stay ≥14 days	<0.001	35.139	8.253	149.616
DNR	0.010	0.352	0.159	0.777
Reoperation	0.008	16.379	2.056	130.483
Model 2				
DNR	0.001	0.086	0.019	0.382
Complexity of surgery ≥6	0.001	4.273	1.776	10.285
Urgency operation	0.073	1.849	0.945	3.616
Model 3				
Length of stay ≥14 days	<0.001	38.016	8.953	161.423
ICU stay	0.012	3.675	1.327	10.177
DNR	0.002	0.294	0.136	0.634

*DNR* do not resuscitate

Thus, nine significant independent triggers were included in the final trigger tool, containing as follows: emergency procedure, complexity of surgical procedure above class 6, do not resuscitate policy (DNR), ICU-stay, length of hospital stay of more than 14 days, reoperation, esophagectomy, Whipple procedure, and acute (or ruptured) abdominal aortic aneurysm surgical procedure.

**Fig. 2:**
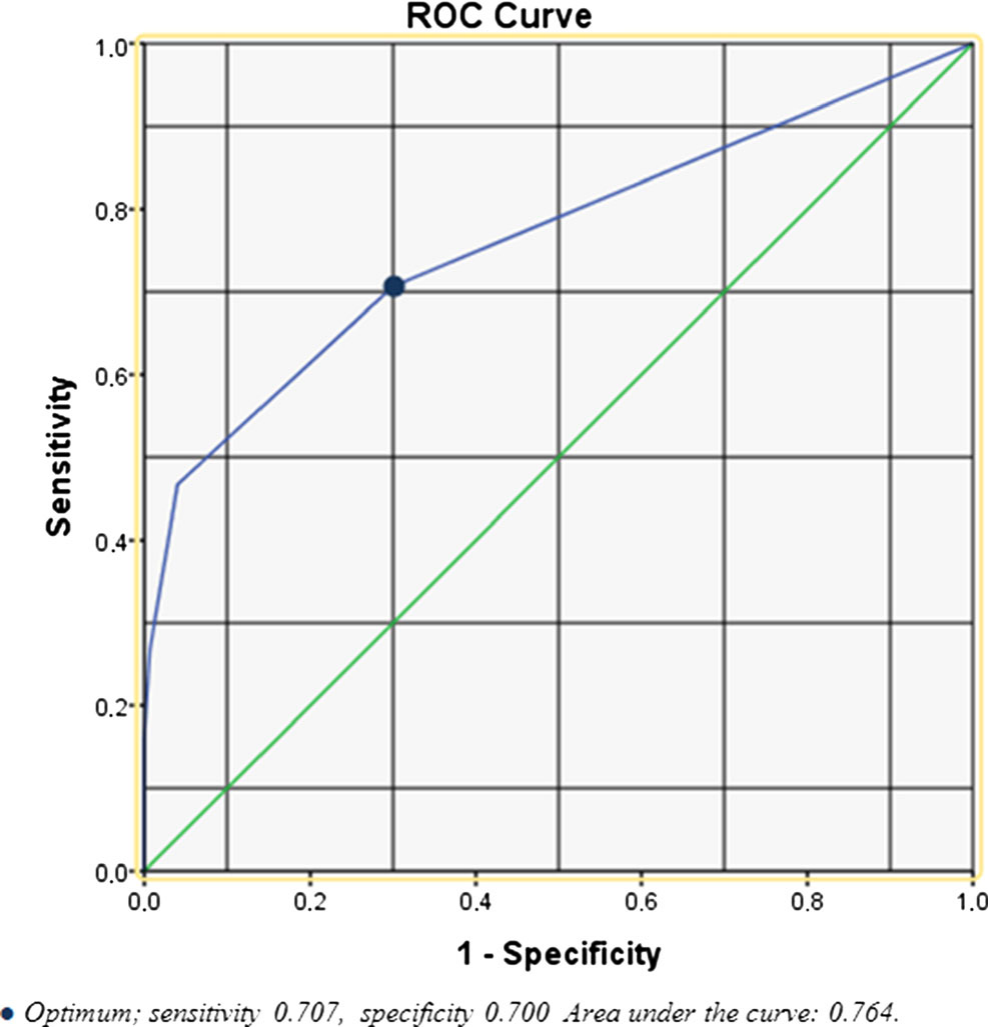
ROC curve of the number of positive triggers needed to detect complications

Receiver operating characteristic (ROC) curve analysis was subsequently performed to determine the number of positive triggers needed to detect complications most accurately. The trigger tool performed best already if one out of the nine triggers in the trigger tool would be present (sensitivity 70.7 %, specificity 70.0 %; AUC 0.764, Fig. 2).

### Comparing verbal inventory with trigger tool

#### Patient records

The sensitivity values of the verbal inventory and trigger tool methods to detect complications as compared to the reference standard were 78.7 % (118/150) and 70.7 % (106/150), respectively, while specificity values were 100 % (150/150) and 70.0 % (105/150), respectively. Hence, the verbal method would miss 21.3 % of the records with complications, while the trigger method would miss 29.3 % (Table 3).

The sensitivity to detect records with major complications (severity class ≥ 2; reoperation,) was higher for the trigger tool than for the SCR, 97.2 and 80.6 %, respectively (Table 4).

**Table 4:** Verbal inventory versus trigger tool, sensitivity. Study group N = 300

	Verbal inventory	Trigger tool
Reference standard		
Records with complication(s) (sensitivity)	78.7	70.7
Records with complication(s) with the highest severity^a^ (sensitivity)		
≥Severity 2	80.6	97.2
≥Severity 3	91.7	100
≥Severity 4	83.3	100

^a^Severity (2) recovery after (re)operation; (3) (probably) permanent damage or function loss; and (4) death

If a combination of the trigger tool and the verbal inventory was used, 138 out of the 150 records with complications would be detected (sensitivity 92.0 %).

#### Missed complications

The verbal inventory missed 31 records with one or more complications (in total 71 complications). The trigger tool missed 45 records (in total 53 complications). All complications missed by the trigger tool were minor complications (severity class < 2), especially wound problems. The verbal inventory also missed mainly minor complications, but these were categorized as functional disturbance (i.e., hypertension or electrolyte derailment). Two severe complications were also missed by the verbal inventory (reoperation and death).

## Discussion

Based on our study results, the proposed trigger tool appears as accurate as a verbal inventory in terms of sensitivity and specificity as to the detection of complications that occur in hospitalized surgical patients. Furthermore, the trigger tool we developed detected a higher number and a higher proportion of more severe complications. Only some mild complications would have been missed, for example, wound infection with no need for a reoperation or cardiac complications.

On the other hand, the verbal inventory during the morning hand-off provides awareness of the complications suffered by their patients and deliberation and reflection with and by their colleagues. The two methods were tested with regard to their ability to identify records with complication(s). To optimize the registration of complications, the results must be referred to the surgeons, who should discuss these in order to undertake preventive actions in, for example, separate complication meetings.

This study is one of the few that used a reference standard to assess the comprehensiveness of the detection of patients with complications by either method. Most studies have used a “silver” standard or even no reference standard at all [13, 14, 18]. The sensitivity of the trigger tool in this study compares favorably to other forms of term searching tools, such as scanning the discharge letters for words suggestive for complications [13, 18], or a natural language processing detecting method [14].

As an alternative to the detection methods investigated here, the clinical observation method is the investigation of potential complications by a trained observer of all patients and providers, who is alerted by a predefined list of clinical event “triggers.” Clinical observation is a powerful tool for identifying incidents and errors in medical care, especially when compared with self-report or voluntary reporting mechanisms [14, 19]. This observation method, however, uses clinical observers and also focus groups to identify complications, which would imply a huge manual effort. Another option, the IHI collaborative “Global Trigger Tool” (GTT), appears to detect more complications than other conventional approaches but requires substantial manual effort [3, 6, 7].

The proposed trigger tool (tailored to a Dutch hospital context) is expected to be highly resource-intensive and will require manual database searching by the database manager whereas the surgeons’ workload will probably minimize. Further research is needed to determine the time and costs. A fully electronic database including all admitted patients and their health care utilization characteristics would facilitate the use of this trigger tool. Unfortunately, symptoms, diagnoses, and physical findings are usually recorded as narrative texts, but are yet unavailable in coded form. Nursing files and surgical discharge letters were found very helpful to find complications [5]. The trigger tool method could be simplified by the retrieval of complications from patient nursing files and surgical discharge letters. In addition, nurses might play an important role in the process of complication registration. Application of the trigger tool should also be simplified by systematized electronic storage information in hospital data systems, in order to detect patients “at risk” more easily.

### Limitations

The study is a single-center study. However, the predictive factors used as potential triggers were derived from a systematic review based on studies from various institutions. Even if not every trigger (for example, highly specialized surgical interventions performed only in high-volume centers) is relevant in some centers, the remaining ones may well be. Three of the triggers equal three major surgical procedures in a university hospital. These procedures are not undertaken in all hospitals. But there has been a tendency for health systems to rush towards inadequately justified “one fits all” solutions [13], but the effectiveness of these global tools vary widely and does not provide the robust data hospitals need to base their decisions on to optimize patient safety. However, the absence of certain triggers or surgical procedures in some hospitals does not imply this trigger tool is invalid. The use of a customized set of triggers to detect surgical complications, as provided in this study, could result in high sensitivity, especially for the detection of major complications. The potential triggers found in a previous systematic review can be used as a set of potential triggers [17], and this article provides the method for other hospitals to develop a customized trigger tool based on their own type of surgery.

Furthermore, the definition of the DNR status may be culture-sensitive. We do not, however, think this will have a major impact on the validity of the trigger tool, but only on the predictive power of this trigger only.

Although tested on a reliable reference standard (*n* = 300), little is known about the risks of the trigger tool in a larger population regarding missed complications. For practical reasons, the number of patients in the reference standard was limited, which may have led to fewer significant triggers. Further external validation is warranted to assess the value of this trigger tool before it can be implemented in clinical practice. The reference standard datasets are a subgroup of departments’ complication database. This procedure might not be statistically appropriate but incorporates only 3 % of the total patient population. Therefore, we assume the influence will be small.

Cutoff values were based on practical grounds and clinical relevance. Determining cutoff values by means of ROC analysis would have resulted in identifying more than 70 % of patient (records) that might have suffered a complication (e.g., the optimum cutoff value for age would be >57, while the patients’ mean age was 55).

This trigger tool is very helpful after discharge in the detection of complications by identifying high-risk patients’ records. Another use of “triggers” could be their functioning as so-called red flags, highlighting the patients who are sensitive for developing complications, which could be useful in the improvement of complication prevention in clinics. Not all of our triggers could function as red flags before admission. The type of surgery and planned IC admission are known before admission. Nevertheless, if these red flags occur during admission, the patient can be marked as “high risk” for developing complications, either to detect them early or even to take actions to prevent (more) complications during admission. Although studies on the GTT are common, little is known about the use of a customized trigger tool [16] or sensitivity outcomes against a reference standard. The need for an efficient and inexpensive means to detect complications makes further research on an electronic (trigger tool) approach attractive.

## Conclusion

The use of a customized set of triggers as proposed here for a university hospital to detect surgical complications results in high sensitivity for the detection of major complications in an academic hospital. On the other hand, mild complications would be missed, for example, wound infection with no need for a reoperation. The proposed trigger tool appears as accurate as a verbal inventory in terms of sensitivity and specificity as to the detection of minor and major complications. This study provides the method for other hospitals to develop a customized trigger tool based on their own type of surgery to detect more severe complications, provided that it is simplified by a systematized electronic storage of patient characteristics and trigger valuable information in hospital data systems.

## Appendix

**Table 5:** Appendix: definition of triggers. Complete list of trigger definitions in alphabetical order used in univariable analysis (Table 1)

Trigger	Definition
Age	Amount of years between date of birth and final hospitalization date on surgical department, round off downwards
Decreased serum albumin	Yes: level of lowest measured serum albumin on day of hospitalization with cutoff level <35 g/L No: level of lowest measured serum albumin on the day of hospitalization which does not reach the cutoff level
Active alcohol abuse	Yes : “active” No: “not applicable” or “previous drinker” Inclusion criteria: When double different registrations for one patient number (different outcomes at multiple registration moments) were determined, outcome closest to admission date were included
ASA score	Fitness of a patient right before a procedure classified according to “Physical Status Classification System” ASA physical status 1—a normal healthy patient ASA physical status 2—a patient with mild systemic disease ASA physical status 3—a patient with severe systemic disease ASA physical status 4—a patient with severe systemic disease that is a constant threat to life ASA physical status 5—a moribund patient who is not expected to survive without the operation ASA physical status 6—a declared brain-dead patient whose organs are being removed for donor purposes Included: the first registered ASA score during a hospitalization on surgical department
BMI	Weight in kilograms divided by the square of length in meters
COPD/asthma/emphysema	Yes: “light”, “medium,” or “severe” No: “no” or “unknown” Inclusion criteria: When double different registrations for one patient number (different outcomes at multiple registration moments) were determined, outcome closest to admission date were included
Increased serum creatinine	Yes: level of highest measured serum creatinine on the day of admission with the cutoff levels >95 μmol/L (women) and >110 μmol/L (men) No: level of highest measured serum creatinine on the day of hospitalization which does not reach the cutoff levels
Increased C-reactive protein (CRP)	Yes: level of highest measured serum CRP on the day of hospitalization with the cutoff level >5 mg/L No: level of highest measured serum CRP on the day of hospitalization which does not reach the cutoff level
Highest urgency code in admission period	Highest urgency code registered during hospitalization S1: direct urgency S2: urgency, procedure occurred during the same part of the day S3: semi-urgency, procedure within 24 h
Hypertension	Yes: “yes, with medication”/“yes, with diet”/“yes, not regulated” according to PDMS database No: “no” or “unknown” according to PDMS database Inclusion criteria: When double different registrations for one patient number (different outcomes at multiple registration moments) were determined, Outcome closest to admission date were included
Admission to IC	Yes: IC admission during hospitalization at surgical department No: no IC admission during hospitalization at surgical department
Length of stay	Difference in days between admission date and final date of hospitalization + 1
Increased leukocyte count	Yes: level of highest measured serum leucocytes on the day of hospitalization with the cutoff level >10.5 × 10^9^ cells/L No: level of highest measured serum Leukocytes on the day of hospitalization which does not reach the cutoff levels
MET score	Fitness score of a patient registered on the basis of a questionnaire including questions on activities which could still be performed by the patient Inclusion criteria: - A registered MET score within a maximum of 30 days before admission date - The most recent MET score registered before admission date MET score 0–9 point
DNR	Yes: resuscitation = - Code A - Code C when “resuscitation yes” No: no resuscitation = - Code D - Code C when “resuscitation no” Inclusion criteria: - Most recent registered resuscitation code according to AMC resuscitation protocol Code A: no treatment limitations Code B: no treatment limitations, permission is required everyday (this code did not appear in our database) Code C: treatment limitations. When is patient is Code C classified further specification of treatment is registered. Resuscitation could be registered as “yes” or “no” Code D: no treatment
Reoperation	Yes: reoperation within - Same hospitalization period - 30 days after discharge date Inclusion criteria: - Reoperation at the same location of previous action Or - Operating a situation which results from previous intervention No: no reoperation
Smoking	Yes: “active” smoker according to PDMS database No: “not applicable” or “previous smoker” according to PDMS database Inclusion criteria: When double different registrations for one patient number (different outcomes at multiple registration moments) were determined, outcome closest to admission date were included
Sodium level outside reference range	Yes: level serum sodium measured on the day of admission most extreme outside reference area. Cutoff levels sodium outside reference area: - <135 mmol/L - >145 mmol/L No: level serum sodium between reference area. Reference area: - 135–145 mmol/L
Use of corticosteroids	Yes: one or more corticosteroid prescriptions known at the AMC pharmacy within 42 days before admission date or at admission date. *No* discrimination between different kinds of steroids products or using period. No: no corticosteroid prescriptions known
Surgical procedure	Yes: surgical procedure during admission performed by a surgeon from the department of surgery. This surgical department provides general, gastrointestinal, hepatopancreatobiliary, vascular, and trauma surgical care No: no surgical procedure during admission
Time required above the scheduled procedure time	Difference in minutes between planned time in OR for surgical procedure and realized time in OR. Yes: difference in minutes between planned time in OR for surgical procedure and realized time in OR No: difference in minutes between planned time in OR for surgical procedure and realized time in OR
Type of procedure: AAAA	Yes: admissions which included a procedure for ruptured or symptomatic abdominal aortic aneurysm Operation code (Dutch Hospital Data^a^): 333535, 333530, 333538, 333153T, 333530H Exclusion: - Elective procedures - Acute rupture thoracic aneurysm - Duplicates The following procedures were checked on inclusion criteria: 333538, 333153T No: admissions with no AAAA procedure
Type of procedure: multidamage control surgery	Yes: admissions on trauma department at AMC hospital with ISS score ≥16 No: admissions with no ISS score or ISS score <16
Type of procedure: esophagus resection	Yes: admissions which included a resection of the esophagus procedure Operation code (Dutch Hospital Data) 334345, 334322, 334327 No: admissions with no esophagus procedure
Type of procedure: Whipple	Yes: admissions which included a Whipple procedure Operation code (Dutch Hospital Data) 335417, 335417A, 335430 No: admissions with no Whipple procedure
Urgency code at moment of admission	Yes: a procedure with urgency code S1/S2/S3 at the day of admission S1: direct urgency S2: urgency, procedure occurred during the same part of the day S3: semi-urgency, procedure within 24 h No: a procedure with no S urgency or no procedure at the day of admission
Urgency code during hospitalization	Yes: a procedure with urgency code S1/S2/S3 during hospitalization S1: direct urgency S2: urgency, procedure occurred during the same part of the day S3: semi-urgency, procedure within 24 h No: a procedure with no urgency code during hospitalization
Complexity procedure	Highest registered complexity of a procedure within a hospitalization Weight class range: 1–7

^a^ www.dutchhospitaldata.nl
